# Molecular Detection of *Dirofilaria immitis* Specific Gene from Infected Dog Blood Sample Using Polymerase Chain Reaction

**Published:** 2017

**Authors:** In Young OH, Kyung Tae KIM, Ho Joong SUNG

**Affiliations:** 1. Dept. of Biomedical Laboratory Science, College of Health Sciences, Eulji University, Seongnam-si, Gyeonggi-do, Korea; 2. ALPHAGENE Co., Ltd., Singu University, Business Incubation Center, Seongnam, Korea; 3. Dept. of Senior Healthcare, BK21 Plus Program, Graduate School, Eulji University, Daejeon, Korea

**Keywords:** *Dirofilaria immitis*, *COI*, Molecular diagnosis, PCR

## Abstract

**Background:**

*Dirofilaria immitis*, a filarial nematode, is the most important parasite-affecting dogs, causing cardiopulmonary dirofilariasis. Current diagnostic tools for detecting *D. immitis* include morphological assays, antigen detection, and X-ray. Herein, we developed a method for the molecular detection of *D. immitis* in blood using polymerase chain reaction (PCR).

**Methods:**

The study was conducted at Eulji University, Republic of Korea in 2016. To detect *D. immitis*-specific gene regions, we aligned the cytochrome c oxidase subunit I (*COI*) genes of seven filarial nematodes and designed primers targeting the unique region. We used dog glyceraldehyde-3-phosphate dehydrogenase (*GAPDH*)-targeted primers as the internal control. We conducted PCR-amplified genomic DNA from canine blood samples. The products were confirmed by sequencing.

**Results:**

Gene alignment revealed a *D. immitis COI*-specific gene region, and the activity of designed primers was confirmed by PCR and sequencing. Plasmid DNA made from the PCR products was a positive control. The limit of detection for our method was 50 copies. The *D. immitis COI* and dog *GAPDH* genes could be discriminated from blood samples simultaneously.

**Conclusion:**

This study provides a method for highly specific and sensitive molecular diagnosis of *D. immitis* used as a diagnostic and therapeutic tool from the early stage of infection.

## Introduction

*Dirofilaria immitis* is a filarial nematode that causes heartworm disease in canines, felines, various wild mammals, and some human populations with increasing incidence in tropical, subtropical, and some temperate regions (1–6). Mosquitoes are the most important vectors for accidental infection of *D. immitis*. When infected mosquito bites a susceptible animal, the third-stage (L3) larvae of *D. immitis* in the head of the mosquito are transmitted to the new final host. After infection, the larvae grow in the blood to the adult stage and subsequently are transferred to the heart of the host, where they cause pulmonary dirofilariasis (1, 2, 4, 7, 8).

Current methods for diagnosis of *D. immitis* include microscopy-based morphological as-says, immunochromatographic antigen detection or ELISA, and X-ray imaging of the main pulmonary artery and the right side of the heart. Methods for molecular detection by PCR are also under development (7, 9–11). Among these diagnostic tools, the most widely used method is microscopic morphological examination of microfilariae from blood samples. However, the number of circulating microfilariae does not correlate with the number of adult heartworms and therefore does not indicate disease severity. Furthermore, this approach has limited sensitivity, and expert analysis is required to distinguish among filarial parasite species because of their rather similar morphology (7, 12). In addition, antigen detection targets the antigens released from the reproductive tract of adult female worms and can give false-negative results during the first 5–8 months of infection due to low worm counts, immature infections, and all-male infections (8,10). Therefore, the development of molecular diagnostic techniques for early diagnosis and therapeutic monitoring is essential, and identification of a sensitive diagnostic molecular marker for heartworm infections is crucial for controlling the disease (7, 13).

This study aimed to develop a PCR-based molecular detection method targeting a *D. immitis*-specific gene in the peripheral blood of infected dogs.

## Materials and Methods

### Identification of the specific gene region of *D. immitis*

The study was conducted at Eulji University, Republic of Korea in 2016. To search for the specific gene region of *D. immitis*, the seven filarial cytochrome c oxidase subunit I (*COI*) gene sequences available were multiple aligned using the Align Sequences Nucleotide BLAST (Basic local alignment search tool) program (NCBI); https://blast.ncbi.nlm.nih.gov/Blast.cgi?PROGRAM=blastn&PAGE_TYPE=BlastSearch&LINK_LOC=blasthome. At the web page, enter the accession number of *D. immitis COI* (EU159111.1) in the Query Sequence box, and then enter the accession number of *Setaria tundra COI* (AJ544874.1), *Setaria digitate COI* (EF174428.1), *Brugia malayi COI* (AJ271610.1), *Wuchereria bancrofti COI* (AJ271612.1), and *Onchocerca volvulus COI* (AM749284.1) in the Subject Sequence box.

**Fig. 1:**
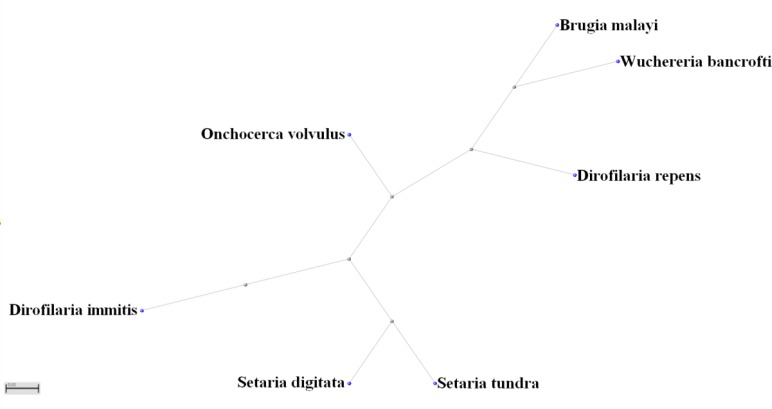
BLAST tree of filarial nematode *COI* genes

The multiple alignments can be obtained by clicking the [BLAST] button at the bottom of the web page. The genome sequences of COI genes of filarial nematodes were obtained from GenBank. Compared with *D. immitis COI*, the six other filarial *COI* gene sequences showed nucleotide identities as follows; *D. repens COI*, 90%; *S. tundra COI*, 89%; *S. digitate COI*, 88%; *B. malayi COI*, 85%; *W. bancrofti COI*, 86%; and *O. volvulus COI*, 89%. The BLAST tree of these results is presented in Fig. 1.

The BLAST tree was created by clicking the [distance tree of result] button at the bottom of the result page of multiple alignments and using the Radial Tree layout.

The BLAST phylogenetic tree for the COI gene was generated using in silico coverage analysis on the NCBI website. The identities (%) of these species compared with D. immitis are reported in the text.

### Design of primers

**Fig. 2:**
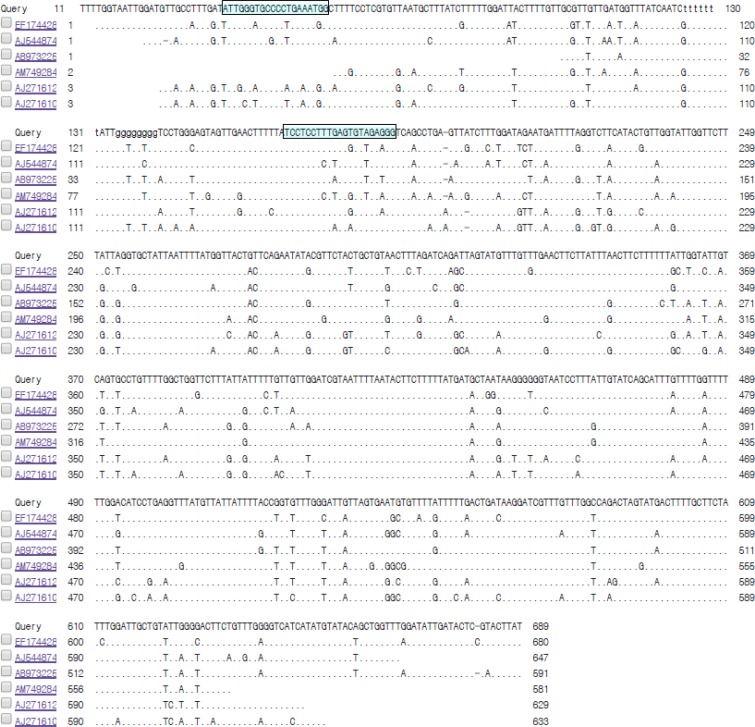
Primer design based on alignment results

The genome sequences of *D. immitis* and *Canis lupus familiaris* (dog) were obtained from GenBank. Based on the multiple sequence alignment described above, primers were designed to target the *D. immitis*-specific gene region (Fig. 2).

Primers targeting specific gene regions were designed based on a highly conserved region of the *COI* gene of *D. immitis* to amplify a 150-bp fragment (forward: ATT GGG TGC CCC TGA AAT GG; reverse: CCC TCT ACA CTC AAA GGA GGA) and the glyceralde-hyde-3-phosphate dehydrogenase (*GAPDH*) gene of dog to amplify a 106-bp fragment (forward: CAT GTT TGT GAT GGG CGT GAA; reverse: GAT GAC TTT GGC TAG AGG AGC). The primer specificities were evaluated using BLASTN 2.3.1+ on the NCBI website. All primers used in this study were synthesized by Integrated DNA Technologies (Coralville, IA, USA).

The boxed region in the top line shows the forward primer sequence of the *D. immitis COI*, and that in the second line shows the reverse primer sequence of the gene. The query was *D. immitis COI* (GenBank: EU159111.1), and the subjects are *Dirofilaria repens COI* (AB973225.1), *Setaria tundra COI* (AJ544874.1), *Setaria digitate COI* (EF174428.1), *Brugia malayi COI* (AJ271610.1), *Wuchereria bancrofti COI* (AJ271612.1), and *Onchocerca volvulus COI* (AM749284.1).

### Preparation of genomic DNA from dog blood samples

*D. immitis*-infected blood samples isolated from random infected source dogs were gifted from Seoul National University, Republic of Korea.

The samples were from Beagles and collected in Jul 2015. Blood samples from healthy volunteers and uninfected dogs that received a routine complete blood count (CBC) test in Veterinary Medical Teaching Hospital of Seoul National University were used as negative controls. Genomic DNA from blood samples collected in EDTA (ethylenediaminetetraacetic acid) tubes was extracted using the QIAamp DNA Mini Kit (Qiagen, Hilden, Germany) according to the manufacturer’s protocol. The concentration of genomic DNA was determined with a NanoDrop spectrophotometer (ThermoFisher Scientific, Sunnyvale, CA, USA).

### PCR amplification

The PCR mixture was prepared with 1× Master Mix (Alphagene, Republic of Korea), 50 ng genomic DNA, and 500 nM primers. The PCR protocol included an initial denaturation step at 95 °C for 5 min; 35 cycles of denaturation at 95 °C for 20 sec, annealing at 60 °C for 20 sec, and extension at 72 °C for 40 sec; and final elongation for 7 min at 72 °C. All amplification reactions were performed on a Veriti^TM^ Dx 96-well Thermo Cycler (Applied Biosystems, Foster City, CA, USA) in a total volume of 20 μL. PCR products were analyzed by electrophoresis on 2% agarose gels.

### DNA sequencing and cloning

To confirm primer activity, the PCR products were sequenced (Bionics, Republic of Korea). After confirmation of the sequences, the PCR products were purified and inserted into pLUG-Prime^®^ TA-cloning vectors (iNtRON Biotechnology, Republic of Korea). The plasmid DNA (pDNA) was cloned and used as a positive control for the PCR reaction. The sequencing results are presented in Table 1.

**Table 1:** Sequencing results

***Description***	***Query cover***	***E value***	***Ident***	***Accession***
1 *Dirofilaria immitis* isolate HU11 cytochrome c oxidase subunit I (COI) gene, partial cds; mitochondrial	100%	2E-69	100%	KM452920.1
2 Canis lupus familiaris glyceraldehyde-3-phosphate dehydrogenase (GAPDH), mRNA	100%	1E-45	100%	NM_001003142.2

### Dual PCR amplification

To detect *D. immitis COI* and dog *GAPDH* simultaneously, the working concentrations of the primers were optimized. The dual PCR condition was the same as that described above.

## Results

### Primer activity confirmation

**Fig. 3:**
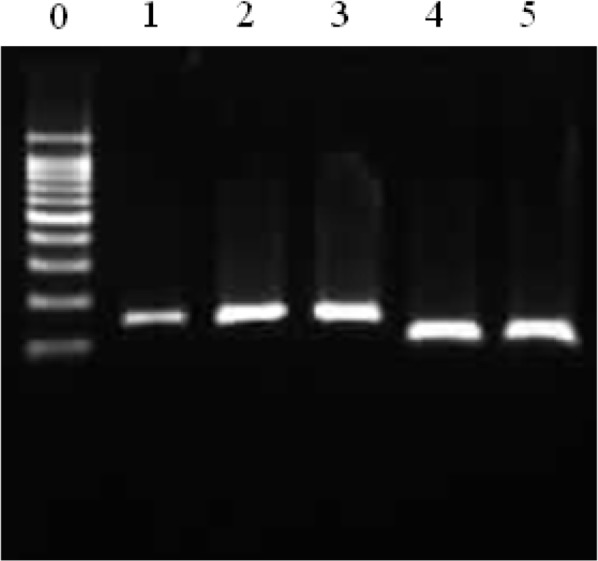
PCR products of plasmid DNA

To confirm the activity of primers, dog blood samples were PCR amplified with the primers, and the PCR products were sequenced. The sequences of the PCR products exactly matched those of the *D. immitis COI* and the dog *GAPDH* gene regions (data not shown). To use the amplicons as positive controls, pDNAs were constructed and cloned using TA-cloning vector. The pDNA was thereafter sequenced to verify the insertion and amplified using PCR (Fig. 3).

Lanes 1–3 (150 bp) show the insert of the *D. immitis COI* pDNA, and lanes 4–5 (106 bp) show that of dog *GAPDH*. Lane 0: 100-bp DNA ladder.

### Limit of detection

**Fig. 4:**
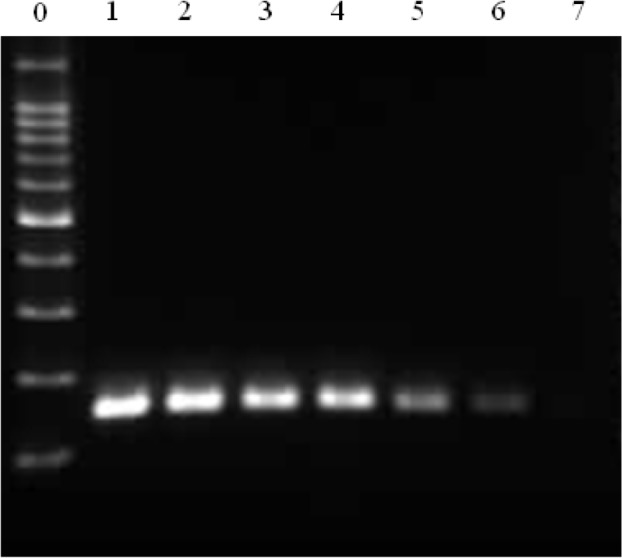
Limit of detection of *D. immitis* DNA

To determine the limit of detection, *D. immitis* pDNA was serially diluted from 5×10^7^ to 5×10^1^ copies and detected by PCR (Fig. 4). The DNA copy number was estimated from the calculated molecular weight of the *D. immitis* pDNA:

$$\mathrm{copy}\;\mathrm{number}=\frac{\mathrm{amount}\;\mathrm{of}\;\mathrm{dsDNA}(\mathrm{ng})\times 3.0221\times 10^{23}(\mathrm{molecules}/\mathrm{mole})}{\mathrm{length}\;\mathrm{of}\;\mathrm{dsDNA}\times 660(g/\mathrm{mole})\times 1\times 10^{9}(\mathrm{ng}/g)}$$

The detection limit was ≤50 copies.

Lanes 1–6: *D. immitis* pDNA; lane 7: distilled water. Ten-fold serial dilutions from 5×10^6^ to 50 copies were employed for lanes 1–6, respectively. Lane 0: 100-bp DNA ladder.

### Optimization of primer working concentrations

**Fig. 5:**
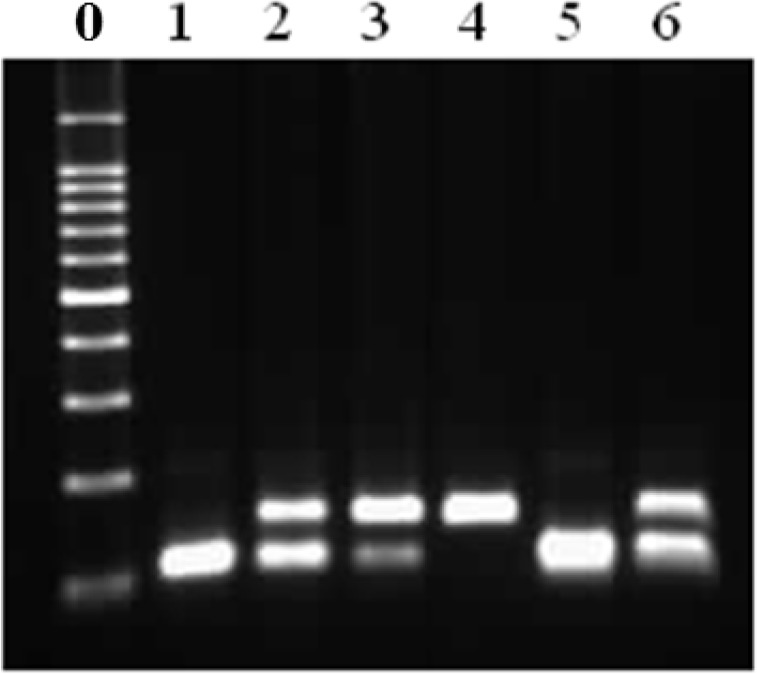
Optimization of primer working concentrations

To amplify *D. immitis COI* gene and dog *GAPDH* gene in the same tube simultaneously, the working concentrations of the primers were optimized (Fig. 5).

The optimized primer concentration ratio was 2:1 (500 nM *D. immitis COI* primers to 250 nM dog *GAPDH* primers). The other concentration ratios yielded strong amplification of one gene but weak amplification of the other due to competition between the two primer sets.

Lanes 1–3: products of dual PCR; lane 4: D. immitis COI (500 nM primers); lane 5: dog GAPDH (500 nM primers); lane 6: combined products (1:1) from lanes 4 and 5. The primer concentration ratios (D. immitis COI to dog GAPDH) were 1:1 (lane 1), 2:1 (lane 2), and 4:1 (lane 3).

### Molecular detection of D. immitis from dog blood samples

**Fig. 6:**
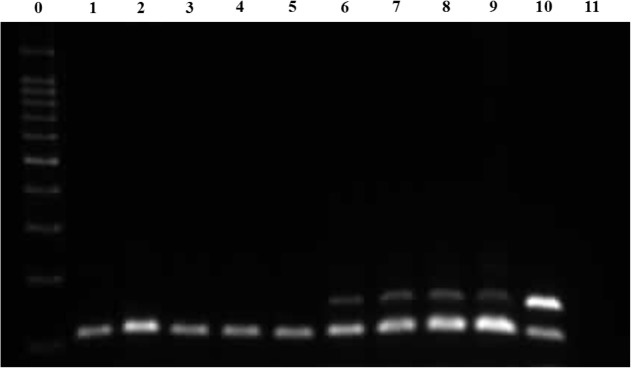
Molecular detection of *D. immitis* using dual PCR amplification. Lanes 1–4: *D. immitis*-negative samples (gDNA from healthy dog blood); lane 5: *D. immitis*-negative control (dog *GAPDH* pDNA as an internal control of *D. immitis* detection); lanes 6–9: *D. immitis*-positive samples (gDNA from infected dog blood); lane 10: positive control (*D. immitis COI* pDNA and dog *GAPDH* pDNA); lane 11: NTC (no template control)

Using the optimized dual PCR conditions, genomic DNA extracted from dog blood samples was amplified. *D. immitis COI* pDNA was used as positive DNA and dog *GAPDH* pDNA was used as an internal control DNA of the assay. *D. immitis COI* pDNA (5×10^6^ copies) and dog *GAPDH* pDNA (5×10^5^ copies) were used positive control templates; dog *GAPDH* pDNA (5×10^5^ copies) was used as the negative control template for the *D. immitis* detection assay. In PCR with *D. immitis COI* and dog *GAPDH* primers, the products of *D. immitis* negative control and negative samples showed only the band for dog *GAPDH* (106 bp), whereas the products of positive control and positive samples showed two bands for *D. immitis COI* (150 bp) and dog *GAPDH*; the NTC (no template control) had no band (Fig. 6).

## Discussion

A direct relationship was reported between *D. immitis* larval development in the mosquito and temperatures in the range of 18–34 °C, indicating that ambient temperature is the major limiting factor in *D. immitis* transmission in most localities (1, 14). The dog blood samples used in this study were collected in the summer in a high-temperature climate (Republic of Korea). In the Republic of Korea, many people breed pet dogs and administer heart-worm medication with or without infection to prevent death due to cardiopulmonary dirofilariasis. However, drug use without pre-diagnosis can lead to development of antibiotic resistance (15). Therefore, a method for accurate and accessible analysis is needed. However, current widely used diagnostic tools have many limitations. To overcome these limitations and improve clinical pathogen detection, we developed a molecular diagnostic tool in this study.

To develop this method, we focused on the mitochondrial *COI* gene. Mitochondrial DNA (mtDNA) genes have high copy numbers, allowing recovery of large amounts of mtDNA from trace samples compared to nuclear DNA.

MtDNA is maternally inherited. It generally does not undergo recombination; thus, its sequence is identical for all maternally linked relatives, and transmission of mtDNA is consistent across many generations. As a mtDNA gene, *COI* is the so-called “barcode” for identifying the last indicator digit of species diversity. *“COI* may be matched by other mitochondrial genes in its efficacy in resolving such cases of recent divergence. This gene is more likely to provide deeper phylogenetic insights than alternatives such as cytochrome b because changes in its amino acid sequence occur more slowly than those in other mitochondrial genes “(16–20).

In this study, we first identified a *D. immitis-*specific region of *COI*. The sequence identity among *COI* genes from filarial species was 85%–90%, with some sequence regions exhibiting complete identity; however, other regions were rarely the same between species. In the inconsistent region, primers were designed to target specifically the *D. immitis COI*. The nucleic acid-based amplification assays were highly sensitive with a limit of detection of fewer than 50 copies and are expected to be highly specific to the *D. immitis COI*. The assay also included amplification and detection of the dog *GAPDH* gene as an internal control. Using adjusted PCR conditions for the duplex reaction, *D. immitis COI*, and dog *GAPDH* gene could be detected simultaneously. This assay provides an opportunity to discriminate *D. immitis* from infected dog blood samples in the early stage of infection, and our qualitative PCR results are the first step to enabling future development studies.

## Conclusion

This study provides valuable information for future studies of *D. immitis* infection, and it could facilitate investigation of the pathogenesis of *D. immitis*. However, in accordance with the characteristics of end-point PCR, this assay can determine only whether a sample is infected. For more precise therapeutic monitoring, therefore, additional development of the quantitative analysis is needed, and this method warrants further investigation.
